# Headaches and Their Treatment

**Published:** 1909-08-28

**Authors:** Guthrie Rankin

**Affiliations:** Physician to the *Dreadnought* and Royal Waterloo Hospitals


### Hospital Clinics.

### HEADACHES AND THEIR TREATMENT.

By GUTHRIE RANKIN, M.D., F.R.C.P.; Physician to the *Dreadnought* and Royal Waterloo Hospitals.

(A Lecture delivered at the London School of Clinical Medicine.)

There is probably no symptom of ill-health more frequently encountered than headache. It forms part of the picture in the early stages of most of the acute organic disorders; is a frequent accompaniment of chronic disease; and is a characteristic phenomenon in many functional disturbances. Its manifestations are varied, and, in some of its more distinctive types, it constitutes the predominant feature of an illness and guides us unerringly to the correct recognition of its cause. It is uncommon in infancy and old age, and is much more frequently encountered in women than in men. It may be induced by a variety of local conditions, such as disorders of the teeth, disease in the throat, nose, ear or eye; refractive errors; rheumatic or gouty affections of the scalp, muscles or fasciæ; or it may ensue upon an inflammatory or traumatic lesion of the cranium. But most frequently of all it depends upon errors of metabolism consequent upon irregular habits of diet and exercise, or upon the occurrence of constitutional or organic lesions.

It must be apparent that a pain which owns such a multiplicity of causes demands considerable care in its investigation, and that nothing can be more illogical than the blind faith with which the public swallows this, that, or the other headache-cure boldly advertised by the unscrupulous quack as infallible.

Let us glance at the most frequent of all forms of headache—that which results from a toxæmic condition of the blood. A toxæmia may be induced either by poisons introduced from without, or by poisons created within the body. Certain drugs, such as iron, quinine, salicine, and opium; unwholesome food containing ptomaines; alcohol when taken in more than physiological amount; and tobacco excessively indulged, may be mentioned as familiar examples of substances which may, when taken into the body, cause headache. The cure of this form of headache is obvious, and consists in the withdrawal of the poisonous substance which is responsible for its production. When it happens in connection with the legitimate administration of drugs for curative purposes, the headache may often be obviated by their admixture with suitable correctives. Quinine can often be tolerated when combined with hydrobromic acid; opium when associated with belladonna or one of the aperient alkalies; and the salicylates when presented with bicarbonate of potash or aromatic spirit of ammonia. In the case of iron, it is often found that one of the milder preparations agrees perfectly when the more potent varieties of the drug are upsetting. Such useful remedies as the citrate of manganese and iron, the valerianate of iron, the salicylate of iron, the syrup of quinine, strychnine and iron, the citrate of quinine and iron, and the peptonate of iron, may be enumerated as useful examples of this class of drug. In regard to alcohol, the subject of treatment is too large to enter upon here, but it may be mentioned, in passing, that in order to assist the patient to accomplish the total abstinence which, in cases of an established alcoholic habit, is essential, he may be helped by such a prescription as the following:—Extract of hydrastis, two grains; extract of belladonna, one-twelfth of a grain; capsicin, one-eighth of a grain; and strychnine, one-thirtieth of a grain: given in the form of a pill three times a day after meals.

Of the poisons created within the body, apart from visceral disease, those which ensue upon a faulty digestion, excessive alimentation, insufficient exercise with consequent ineffective elimination of waste products, are mainly responsible for headache and other evil consequences. This variety of headache is due primarily to interference with hepatic activity, and to fermentative or putrefactive processes in the gastro-intestinal tract. For its relief the food must be of the simplest and most bland description, and should be carefully adapted to the patient's digestive capacity. In cases where the stomach is dilated and its walls are flabby, a few morning wash-outs through a syphon-tube followed by the application of the faradic current, and twenty minutes massage to the abdominal walls, will be found useful. In patients who have to blame an overnight revel or an unwise evening meal for their headache, the speediest means of relief is afforded by an emetic. In order to stimulate hepatic activity, podophyllin, grey powder, blue-pill, calomel, iridin, or leptandrin, combined with either colocynth or rhubarb should be resorted to. For the prevention of intestinal fermentation, antiseptics are valuable and may be given in an acid or alkaline mixture, according to the indications of the case.

(a) Dilute hydrochloric acid, twenty minims; pure carbolic acid, two grains; strychnine solution, five minims; tincture of ginger, twenty minims; decoction of cinchona-bark to one ounce: to be taken three times a day one hour after meals.

(b) Sulphocarbolate of soda, ten grains; bicarbonate of soda, fifteen grains; tincture of nux vomica, ten minims; spirits of chloroform, twenty minims; compound infusion of gentian to one ounce: to be taken three times a day, one hour before meals.

In cases which come under this category, help is also afforded by the inclusion in the daily dietary of one pint of soured milk. This is conveniently prepared at home by the use of the lactic-acid tablets, put up by Allen and Hanbury under the name of "Sauerin." The proper degree of "souring" is produced in the milk by its treatment in the Sauerin-apparatus, supplied by the same firm, which is sent out with complete directions.

Headache accompanies all acute fevers and inflammatory disorders. It is, as a rule, confined to the earlier stages of the illness and may be allayed by ice or cold-water cloths applied to the scalp, or by a mustard plaster to the nape of the neck, but otherwise its treatment becomes merged in that of the general disorder. Its incidence, duration, and degree vary according to the exciting cause, and it sometimes presents features which, when read into the text of the general condition, help to reveal the disease behind it; as, for instance, in influenza, we find the pain is specially intense in the globes of the eyes, or in enteric fever, where it is often the earliest and most continuously persistent symptom, slight in the morning, but increasing in intensity towards evening. In these instances—and many similar might be quoted—the meaning of the headache is subsequently explained by the evolution of the disorder producing it, but regarded *per se*, its own characteristics often serve, from the beginning, to guide the diagnosis. The susceptibility of gouty and rheumatic people to headache peculiar to their diathesis is not sufficiently recognised. In a patient, proved to be gouty from the experience of one or more attacks of classical great-toe inflammation, we are not surprised to find a history of frequent moderate headaches which yield to a dose of calomel and a temporary application of the muzzle, and we regard such occurrences as the inevitable consequence of a sluggish liver or of some passing dietetic indiscretion. But there is another form of headache to which the gouty are liable, which is of much more serious consequence, and which is not infrequently misinterpreted. The pain is of sudden onset and frequently sets in after a time of unusual stress; it is bi-temporal in situation, throbbing in character, accentuated by movement, accompanied by vertigo on any sudden change of position, and frequently increased during the night. It is mostly met with in men of a full habit of body, and is accompanied by a flushed face; a scanty secretion of high-coloured urine, which may or may not throw down a copious deposit of lithates on standing; nausea and loss of appetite; irregularity of bowels, with abnormally pale stools; mental depression and confusion of thought; and by a small, rapid, high-tension pulse, often associated with palpitation and shortness of breath on exertion. This variety of headache is suggestive of apoplexy, and always demands prompt and active attention.

The rheumatic headache is of quite a different type. It affects the epicranial aponeurosis and the tendinous terminations of muscles. The pain is superficial and causes tenderness of the scalp; it is often specially pronounced over certain circumscribed areas of the vertex, or at the seat of one or more tendinous insertions, where small fibrous swellings are not uncommonly to be felt on palpation. It is worst in the evenings, but is subject to constant variations in intensity, and can be readily excited by movements of rotation of the head. In the headache which belongs to renal disease, the pain is dull, severe, and constant; it occupies the entire forehead, and is accompanied by a sensation of fulness within the head, surging in the ears, dimness of sight, and a tendency to slight delirium, and subsequent drowsiness. Confirmatory evidence of its ætiology is furnished by vomiting and diarrhœa, by the presence in the urine of albumen and casts; sometimes by the existence of retinal changes; and by oppression in the chest and asthma.

The headache which occurs as a prominent symptom of influenza is rapidly relieved by such a prescription as this:—Antipyrin, ten grains; aspirin, ten grains; citrate of caffein, three grains; dispensed in a cachet and given every three or four hours until the pain is relieved. In enteric fever, headache does not yield in the same satisfactory way to analgesic remedies; it is more amenable to chloral hydrate and potassium bromide than to most other drugs. Ten grains of chloral with twenty grains of one of the bromide salts seldom fail to give temporary relief. Bromidia, which is a mixture of chloral, bromide, and cannabis indica, is a useful preparation in many enteric cases; its administration at bedtime often ensures a good night's rest. The headache which so often troubles the person of gouty proclivities ought to be treated on the lines indicated for the management of dyspeptic conditions; but in that form of sudden and severe pain in the head which has been referred to as a specially important incident in patients who have previously suffered from acute gout, more active measures are indicated, and in addition to colchicum, citrate of potash, and the usual anti-gouty remedies, four or six leeches should be applied to the temples, and the bowels ought to be copiously evacuated by a five-grain dose of calomel given at bedtime, followed in the early morning by two teaspoonfuls of Carlsbad salt, repeated every hour until a satisfactory result is obtained. The headache of rheumatism is always relieved by the local application of warmth, and often yields speedily to a combination of chloride of ammonium, twenty grains; salicin, ten grains; and phenacetin, ten grains: given three or four times a day. In renal headache simple diluents should be given freely, and the diet restricted to milk. All the eliminating organs must be stimulated. The skin is most speedily acted upon by pilocarpine given hypodermically in a daily dose of one-sixth to one quarter of a grain, the patient being previously placed in a hot pack where he should remain for an hour. The free action of the kidneys will be promoted by squills, digitalis, spirits of juniper, acetate of potash, or cream of tartar; these failing, success often follows the administration of diuretin in ten grain doses every four hours. The bowels should be excited to purgative action by compound jalap powder in forty to sixty grain doses, or elaterium in a dose of one quarter of a grain, or croton oil in such a pill as this:—Croton oil, one minim; oil of carraway, one minim; extract of colocynth, three grains. When high arterial tension and asthma are obtrusive symptoms, as they often are in advanced cases of interstitial nephritis, their early relief is an urgent necessity. This is sometimes satisfactorily accomplished by the following prescription:—Iodide of potassium, ten grains; the one per cent. solution of nitro-glycerine, two minims; aromatic spirit of ammonia, half a drachm; and chloroform water to half an ounce, to be given every three or four hours.

Another form of headache which demands the constant attention of most of us is migraine—or as otherwise known, on account of its common unilateral distribution, hemicrania. It occurs more often in women than men, is more common on the left than on the right side of the head, and is often hereditary. It is almost as frequent in childhood as in adult life, and generally diminishes or disappears in old age. It is always associated with vaso-motor phenomena, and is frequently accompanied by high arterial tension. It manifests itself in paroxysmal attacks, and is, in many respects, analogous to epilepsy. The two disorders not uncommonly co-exist in different members of the same family. The migrainous attack usually sets in during the early hours of the morning, and is preceded by prodromal warnings such as vertigo, yawning, dancing specks before the eyes, zig-zag patterns, or tinnitus aurum. These sensations correspond closely to the aura that precedes an epileptic seizure. The pain of migraine is accompanied by extreme intolerance of noise or light, and by a supreme desire to be left alone and undisturbed. Finally the attack terminates in a troubled sleep from which the sufferer awakes free of headache, but languid and irritable.

Remedies for the relief of the migraine are almost without number, but the most that can be expected of any of them is a diminution of the severity, and a curtailment of the duration of the pain once the attack has become fairly established. On the first threatenings of an attack, the patient should lie down in a darkened room, and if the cause be immediately antecedent fatigue, ten grains of antipyrin swallowed with one tablespoonful of brandy and water will often, when combined with one or two hours' rest, cut short the pain. In more acute cases such simple measures are insufficient. It is then necessary for the patient to go to bed and to submit to wholesome starvation for twenty-four hours. Primary relief is afforded by the application of cold to the head, and of a mustard-plaster the whole length of the spine. If there is reason to suppose that the stomach contains a quantity of undigested and fermenting food, it should be emptied by an emetic. For the immediate relief of pain there is a long list of analgesic drugs to choose from. I find, in my own experience, one or other of the following combinations most effective:—

(a) Antifebrin, two grains; citrate of caffein, three grains; lupulin, one grain.

(b) Antipyrin, ten grains; aspirin, ten grains; codeine, one quarter of a grain.

(c) Pyramidon, seven grains; dried bromide of strontium, ten grains; valerianate of zinc, two grains.

To be put up in cachet form, and one to be taken every two hours for three doses, or until the pain subsides. Afterwards the doses to be taken at longer intervals.

In cases of extreme severity, when all the remedies of the foregoing class fail, it may be exceptionally necessary to resort to a hypodermic dose of one-quarter of a grain of cocaine or morphia, the latter being most efficacious if given in combination with one-hundredth of a grain of atropin. When the migrainous attack is associated with a pulse of high tension, whatever remedy is chosen should be accompanied by nitro-glycerine in one or two minim doses. Between the attacks of pain something may be done in the way of prevention by proper regulation of the daily life as regards diet, exercise, clothing, occupation, etc.; by keeping the liver active with occasional small doses of calomel and rhubarb; and by administering arsenic and cannabis indica in combination with an intestinal antiseptic.

Closely allied to migraine is yet another variety of headache associated with disturbance of one or other branch of the trigeminal or fifth cranial nerve. The most frequent cause of this neuralgic headache is exposure to cold and damp; but it may also be produced by the irritation of a decayed tooth, by disease in the antrum, or by the pressure of an inflammatory exudation or morbid growth near one of the bony canals traversed by a branch of the nerve. The pain is deep-seated, and of a stabbing and burning character. It may involve any of the three divisions of the nerve, and is always confined to one side of the face. It varies in intensity, but in its more severe manifestations it is accompanied by spasmodic unilateral contraction of the facial muscles, and causes the patient to cry out with the agony he suffers; it is then known as Tic Douloureux. Tender spots along the course of the affected nerve are characteristic, and are most commonly found at the supra-orbital notch, over the infra-orbital foramen, in front of the ear, or at the seat of exit of the inferior dental nerve. Another, but less common, form of neuralgic headache is confined to the occipital region, and is met with when the posterior branches of the first four pairs of spinal nerves are the seat of disturbance. The first indication for treatment is the removal of the cause when this can be ascertained, and is possible to deal with. The ears, mouth, throat and antra must be investigated, and particularly the teeth should be minutely examined, special care being taken to ascertain that a buried stump or a small root-abscess is not primarily responsible for the pain. The local application of sedatives may succeed in relieving the intensity of the suffering. The following applications are useful for this purpose:

(a) Menthol, two drachms; pure chloroform, two drachms; olive oil, one and a half ounces.

(b) Sulphate of atropine, five grains dissolved in one ounce of distilled water.

(c) Liniment of belladonna, liniment of chloroform, aconite and soap in equal parts.

When the pain becomes very acute it will be found necessary to obtain initial relief from one or more subcutaneous injections of morphia, and, to be effective, the dose must be from one quarter to half a grain. Hyoscine is sometimes more successful than morphia. It may be given hypodermically in doses of one two-hundredth of a grain. In this variety of headache gelseminum, which seems to exercise a specific influence upon the peripheral branches of the fifth nerve, should always be administered. It is well to search for some dyscrasial tendency—gouty, rheumatic, malarial, syphilitic, or anæmic—as a guide to the selection of medicaments which may enhance the curative influence of gelseminum, and from the following formulæ that should be chosen which seems best to meet the indications of the case under observation:

(*a*) Citrate of potash, thirty grains; compound tincture of colchicum, twenty minims; tincture of gelseminum, fifteen minims; decoction of taraxacum, to one ounce.

(*b*) Salicylate of soda, fifteen grains; antipyrin, ten grains; tincture of gelseminum, fifteen minims; camphor water, to one ounce.

(*c*) Sulphate of quinine, five grains; hydrobromic acid, half a drachm; tincture of gelseminum, fifteen minims; infusion of orange, to one ounce.

(*d*) Iodide of potassium, ten grains; Fowler's solution, three minims; tincture of gelseminum, fifteen minims; decoction of sarsaparilla, to one ounce.

(*e*) Ammoniated citrate of iron, ten grains; acetate of ammonia solution, one drachm; tincture of gelseminum, fifteen minims; tincture of nux vomica, ten minims; peppermint water, to one ounce.

Any of these mixtures may be taken every four or six hours.

In some intractable cases croton-chloral succeeds better than any other drug. It may be given conveniently in this combination:—Croton-chloral-hydrate, four grains; extract of gelseminum, one-quarter of a grain; heroïn, one twelfth of a grain: in a pill, every three or four hours until relief is obtained.

Recently cases have, from time to time, been recorded of striking temporary relief being obtained by injecting the main trunks of the nerve at their points of emergence from the skull with an 80 per cent. solution of alcohol, according to Schlösser's method. The administrative technique is difficult, requires the assistance of an anæsthetic, and is attended with a considerable degree of subsequent discomfort.

Time will only permit me to refer casually to a few other forms of headache. That which is caused by organic changes affecting either the meninges or the brain, and which accompanies such conditions as meningitis, intra-cranial tumour, abscess, or hæmorrhage, is deep-seated and continuous, is made worse by stooping or exertion, and is markedly increased at night. Its distribution is often frontal, but it is occipital when the cerebellum is the seat of lesion, and may occupy any part of the scalp in an area overlying a cortical lesion. Among the more important accompanying symptoms are vomiting, optic neuritis, vertigo, irregularity of pulse, ocular or other paralyses, convulsive movements, intellectual aberration, and coma. The headache of syphilis is peculiarly given to nocturnal exacerbation: if it moderates during the day, it will increase in severity towards a certain hour of the night and prevent sleep. In meningitis, the pain is diffused over the skull and is accompanied by pyrexia, photophobia, retraction of the head and delirium. In apoplexy, there is almost always a prodromal headache, limited to one parietal or temporal region, and often accompanied by confusion of thought and vertigo. The headache which results from an intracranial growth should be treated initially by iodide of potassium. If the tumour is specific, the iodide may prove completely curative, but it is also capable of relieving, to a certain extent, the pain and local congestion induced by non-specific swellings. It is of importance to remember in connection with the administration of the drug in such cases that, to be effective, the dosage must be large—from thirty to forty or even sixty grains three or four times a day. If treatment by iodide fails, and if the clinical signs enable the situation of the tumour to be localised, the question of possible relief from surgical interference must always be considered. When the headache is due to meningitis, thrombosis, or hæmorrhage, treatment of the pain becomes merged in that of the general condition. The headache of eye-strain dependent upon astigmatism, presbyopia, or glaucoma requires ophthalmoscopic and retinoscopic examination for its diagnosis, and its cure falls within the province of the ophthalmic surgeon.

The headache of neurasthenia is probably due to some form of auto-intoxication, and demands for its relief the treatment described as suitable for toxæmic headaches, plus the system of rest, massage, isolation, and super-alimentation associated with the Weir-Mitchell plan of management. It may be worth mentioning that when these neurasthenic cases are associated, as they so often are, with disturbance of the vaso-motor system, distinct improvement often ensues upon the exhibition of ichthyol, which seems to have a specific influence upon the vaso-motor centres as well as an antiseptic effect upon the gastro-intestinal tract. The following prescription has proved of signal service to me in a large number of such cases: Ichthyol, four grains; valerianate of zinc, three grains; extract of cannabis indica, one-third of a grain; arsenious acid, one-fortieth of a grain; iridin, one grain: in capsules thrice daily.

Another common source of headache is met with in the two opposite vascular conditions of plethora and anæmia. The plethoric headache is that which characterises gouty conditions, or threatened apoplexy, already referred to; but it is also met with at the onset of pyrexial disorders, in certain forms of valvular heart disease, after an epileptic seizure, as a consequence of alcoholic excess, or sometimes in sudden menstrual suppression. The pain is best relieved by cold to the head; temporary abstinence from food; diluents; lactate of calcium, or an alkaline mixture containing bromide of potassium; and temporary rest in bed. In cases where the cerebral vessels are very loaded, the most speedy relief is obtained by venesection or the use of leeches, and by sinapisms applied over the abdominal wall.

The anæmic headache is most frequently vertical, but it often assumes the neuralgic type. All remedies which increase vascular tension, accelerate the circulation through the brain, and improve the quality of the blood are serviceable; of these the most valuable are arsenic, iron, and citric acid which may be ordered in many varieties of combination to meet the requirements of individual patients. Causes which contribute to the anæmia must be dealt with as part of the cure. Anæmic headaches are relieved by alcohol, and its administration in moderate quantity, in the form of a light red wine with luncheon and dinner is often advantageous.

